# The temporal precision of audiovisual integration is associated with longitudinal fall incidents but not sensorimotor fall risk in older adults

**DOI:** 10.1038/s41598-023-32404-y

**Published:** 2023-05-03

**Authors:** Alan O’Dowd, Rebecca J. Hirst, Annalisa Setti, Orna A. Donoghue, Rose Anne Kenny, Fiona N. Newell

**Affiliations:** 1 School of Psychology and Institute of Neuroscience, Trinity College Green, Dublin 2, D02 PN40 Ireland; 2 The Irish Longitudinal Study on Ageing, Trinity College Dublin, Dublin, Ireland; 3 School of Applied Psychology, University College Cork, Cork, Ireland; 4 Mercer Institute for Successful Ageing, St. James Hospital, Dublin, Ireland

**Keywords:** Psychology, Risk factors

## Abstract

Sustained multisensory integration over long inter-stimulus time delays is typically found in older adults, particularly those with a history of falls. However, the extent to which the temporal precision of audio-visual integration is associated with longitudinal fall or fall risk trajectories is unknown. A large sample of older adults (*N* = 2319) were grouped into longitudinal trajectories of self-reported fall incidents (i.e., decrease, stable, or increase in number) and, separately, their performance on a standard, objective measure of fall risk, Timed Up and Go (TUG; stable, moderate decline, severe decline). Multisensory integration was measured once as susceptibility to the Sound-Induced Flash Illusion (SIFI) across three stimulus onset asynchronies (SOAs): 70 ms, 150 ms and 230 ms. Older adults with an increasing fall number showed a significantly different pattern of performance on the SIFI than non-fallers, depending on age: For adults with increasing incidents of falls, those aged 53–59 years showed a much smaller difference in illusion susceptibility at 70 ms versus 150 ms than those aged 70 + years. In contrast, non-fallers showed a more comparable difference between these SOA conditions across age groups. There was no association between TUG performance trajectories and SIFI susceptibility. These findings suggests that a fall event is associated with distinct temporal patterns of multisensory integration in ageing and have implications for our understanding of the mechanisms underpinning brain health in older age.

## Introduction

The typical ageing process can be associated with declines in cognitive health, musculoskeletal function and sensory processing^1,2^. Changes also occur in how the ageing brain integrates multiple sources of sensory information^3^, particularly when there is a temporal delay in stimulation across the senses. That is, while robust multisensory integration in younger adults is typically observed for cross-sensory events that occur close together in time, integration in older adults tends to be sustained over longer inter-stimulus intervals^4,5^. This prolonged integration may, in turn, lead to less precise perceptual judgements in the real world. This has been demonstrated in studies utilising the Sound Induced Flash Illusion (SIFI), a validated measure of temporal audio-visual integration in which audition influences visual perception^6,7^. In this illusion, when one visual ‘flash’ is presented together with two auditory ‘beeps’, observers tend to report seeing two visual ‘flashes’. This is influenced by the reliability of the sensory information as well as prior perceptual experience, in line with Bayesian optimal inference^8^. Studies have shown that illusion susceptibility in older adults is sustained over longer audio-visual asynchronies relative to their younger counterparts^4,5^. This suggests a widening of the temporal binding window, the optimal period of time in which sensory information is most likely to be integrated^5,9^. Previous cross-sectional studies have suggested that imprecise temporal multisensory integration is pronounced for older adults with less healthy cognitive profiles^10,11^ including mild cognitive impairment^12^, as well as decreased gait speed^13^ and a history of falls^14,15^.

Balance and postural control, critical for everyday behaviours such as walking, are informed by multisensory processes, relying on the weighting and combination of signals from multiple sensory systems^1,16^. Indeed, inefficient multisensory integration may predispose an older adult to a fall^17,18^. Other evidence suggests a link between general multisensory integration and balance control. For example, susceptibility to the SIFI is greater in older adults when assuming a standing posture versus sitting^19^ while training on balance function not only improved balance function in older adults but also was associated with a significant reduction in susceptibility to the illusion^20^. As noted by Setti et al.^14^, an expanded temporal binding window in ageing specifically could lead to ‘inefficient’ sensory weighting processes and/or increased distraction from task-irrelevant information, compromising one’s ability to appropriately maintain balance. While previous findings suggest that performance on the SIFI may be useful in discriminating older adults with different health outcomes^10–15^ there has also been some inconsistency in the literature. For example, Setti et al.^14^ reported an overall difference in susceptibility to the SIFI between older adults with a history of falls versus their healthy non-faller counterparts. However, neither Stapleton et al.^19^ nor Merriman et al.^20^ found such a group difference.

When reported, findings of an association between multisensory integration and falling have mainly been demonstrated in cross-sectional studies^14,15^ or across older adults differing in falls status [e.g.,^14^]. In order to help further establish this relationship, longitudinal evidence is required. To that end, here we investigated the relationship between temporal audio-visual integration and *longitudinal* fall numbers or fall risk trajectories in a large sample of older adults. Capturing longitudinal trends in fall incidents and fall risk may be advantageous in shedding light on whether multisensory integration is sensitive to the broad fall profiles of older adults. This is particularly important to capture, given that falling is complex, multidimensional and often difficult to predict^21,22^. As such, fall profiles that are measured at a single time point are unlikely to capture the extent to which an older adult is at risk of further decline or not. In our previous work involving older adults from The Irish Longitudinal Study on Ageing (TILDA)^11^ we demonstrated that susceptibility to the SIFI is sensitive to trajectories of performance on cognitive tasks over (at least) 10 years. Therefore, along with evidence for cross-sectional associations between falls and multisensory integration, it is reasonable to ask if SIFI susceptibility is similarly discriminative of longitudinal physical functioning related to balance control in older adults.

To address this question, we investigated a large sample of older adults drawn from TILDA^23^. In order to comprehensively capture the fall profiles of these older adults, we assessed their self-reported incidents of falls, based on the number of falls reported at each testing wave (from a total of 5 test waves over 10 years), and fall risk which is typically based on performance on the Timed Up and Go task^22,24^, also captured at each wave. We considered it important to independently capture both long-term fall incidents and sensorimotor fall risk given that older adults who have not yet fallen can still experience perturbed mobility while those who have fallen may not necessarily be at high risk of falling again (particularly if the fall incident was a simple slip or trip). As such, the inclusion of both of these measures could help shed important light on whether the precision of temporal multisensory integration is specifically associated with the act of falling itself or more general sensorimotor decline. Furthermore, this dual approach is consistent with the broader literature, as studies of multisensory integration have either recruited older adults with a history of falls to compare with non-fallers or fall risk was determined based on measures of sensorimotor function [for a review see^17^]. We constructed longitudinal trajectories for fall number and sensorimotor fall risk, incorporating data spanning 10 years (five testing waves of TILDA). Accuracy on the SIFI^6,7^, assessed at a single time point (i.e., testing wave 3), was used as a valid and reliable measure of audio-visual integration. If multisensory integration is linked to long-term patterns of falling, we expected that older adults with a higher incidence of falls over 10 years would show less precise temporal multisensory integration (i.e., sustained susceptibility to SIFI at long SOAs) than their healthier counterparts. If multisensory integration is independently linked to long-term patterns of sensorimotor fall risk, then we also expected that older adults with slower TUG times (i.e., reduced mobility and higher fall risk) would show less precise temporal multisensory integration than their healthier counterparts.

## Methods

### Study population

**Figure 1:**
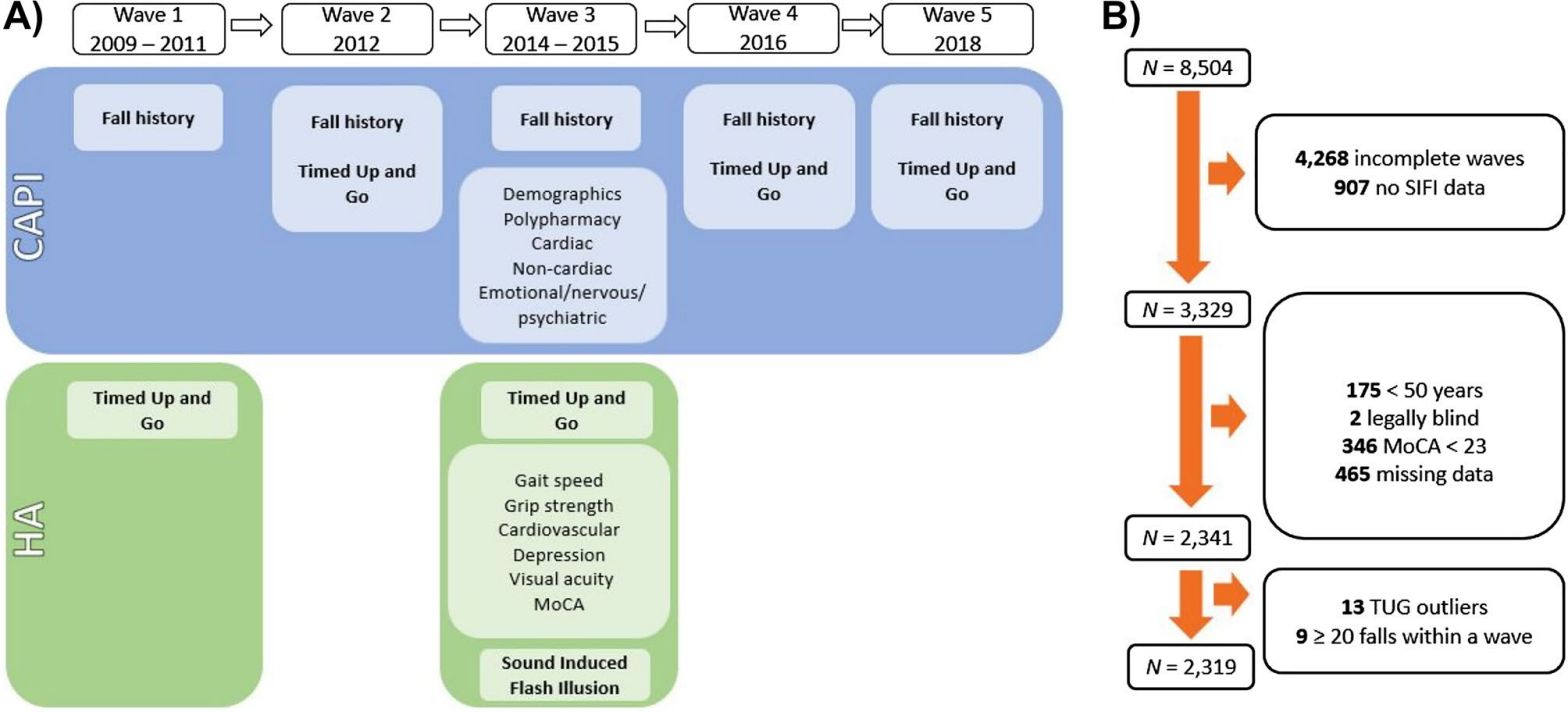
An illustration of the testing protocol on waves 1–5 of TILDA and measures of participant inclusion. (**A**) A schematic summary of the variables included in the present study. Variables shown in bold were used for group determination (fall history and Timed Up and Go) or as the primary outcome measure (Sound Induced Flash Illusion). Other variables were used for exclusion criteria and/or descriptive purposes. CAPI = Computer Assisted Personal Interview. HA = Health assessment. MoCA = Montreal Cognitive Assessment. ‘Cardiac’ includes self-reported doctor diagnosis of stroke, ministroke, heart attack, heart murmur, abnormal heart rhythm and/or angina. ‘Non-cardiac’ includes self-report doctor diagnosis of Parkinson’s Disease, chronic lung disease, asthma, arthritis, osteoporosis and/or cancer. ‘Emotional/nervous/psychiatric’ disorder includes hallucinations, anxiety, depression, schizophrenia, psychosis, mood swings and manic depression. ‘Cardiovascular’ refers to an active stand test of cardiovascular function. (**B**) A schematic summary of the sampling exclusion criteria.

Participants were drawn from waves 1–5 (2009–2018) of TILDA, a population representative sample of 8504 individuals, resident in the Republic of Ireland^23^. The study was approved by the Trinity College Dublin Faculty of Health Sciences Research Ethics Committee and complied with relevant data protection legislation. All participants provided informed consent at every testing wave. Data were excluded prior to analysis, as shown in Fig. 1. Consistent with previous studies involving the TILDA cohort, in which SIFI susceptibility was the outcome measure^10,11,13^, data from participants who either did not partake in all five waves (*n* = 4268), provided no data for the SIFI task (*n* = 907), were younger than 50 years (*n* = 175), were registered as legally blind (*n* = 2), had a suspected mild cognitive impairment (Montreal Cognitive Assessment Score < 23 at wave 3 when the SIFI was conducted; *n* = 346) and/or had missing data for important model predictors/covariates (*n* = 465) were omitted prior to analysis.

### Measures of fall history and fall risk

We constructed longitudinal trajectories based on incidents of falls and fall risk separately. Self-reported number of falls was used as the primary measure of falling, indicating both whether a participant had fallen *(“Have you fallen…”)* and, if so, how frequently fall incidents occurred over the testing waves (“*How many times have you fallen…”)*. The participants were asked to report how many falls they had experienced in the last year (for wave 1) or since the last wave (for waves 2–5). No formal definition of a fall was provided. The Timed Up and Go (TUG) task was selected as the primary measure of sensorimotor fall risk, consistent with previous studies^22,24^. TUG time was assessed by a trained healthcare nurse, in a health centre at waves 1 and 3 and in the participants’ own homes at waves 2, 4 and 5. In the health centre, a chair (height of 46 cm with armrests) was used. In the home environment, an available chair was used that best matched the centre chair. The participants were instructed to walk at their normal pace and walking aids were allowed if required. The participants were timed as they stood up from the chair upon a verbal prompt, ‘GO’, and walked three metres in a straight line to a visually defined marker on the floor before returning to the same chair. Once they were seated again with their back against this chair, the timing was stopped. The TUG task is a clinical measure of functional mobility, reported to assess muscle strength, balance and gait^24,25^ and is, therefore, taken as an objective predictor of fall risk and frailty in older adults^22,24–26^. As our aim was to investigate the association between long-term fall profiles and multisensory integration, and both fall number and TUG times were continuous in nature, we applied longitudinal anchored k-medoids clustering via the ‘akmedoids’ package^27^. Anchored k-medoids is a robust alternative to k-means clustering that allows for the uncovering of directionally homogenous, long-term, linear trends in data, while minimising the influence of outlying values and short-term fluctuations in data over time^27^.

Prior to including measures of TUG in our analyses, we omitted observations from individuals who showed atypically long (> 350 s, which was over 10 SDs above the sample mean at that wave) or short (< 4 s) TUG times over the testing waves (*n* = 13). To mitigate the possibility that falls reflected other serious intrinsic factors, we also omitted data from nine individuals who reported falling 20 times or more at a single wave. This yielded a final sample of 2319 older adults (mean age at wave 3, 64.54 years, *SD* = 7.20; 54% female). Anchored k-medoids clustering^27^ was conducted for this sample for 3–5 clusters using 100 redraws. For fall number, clustering was performed only on those who reported falling at least once over the five testing waves (*n* = 1316), as the older adults who never reported experiencing a fall over 10 years (*n* = 1003) automatically constituted a standalone group and, for the purpose of our analysis, should not be clustered in the same groups as older adults with fall incidents. To determine fall risk, clustering was performed using TUG data from all participants.

### Performance on the sound-induced flash illusion

The precision of temporal multisensory integration was assessed as susceptibility to the Sound Induced Flash Illusion (SIFI) across different Stimulus Onset Asynchronies (SOAs)^6,7^. SIFI susceptibility is widely considered as a valid and reliable measure of the temporal precision of audio-visual integration and is associated with activity in a network of brain regions including the primary visual and auditory cortices, superior temporal sulcus and anterior cingulate^5^. The SIFI was included as part of a comprehensive health assessment only at wave 3 of TILDA.

The task was performed in a dimly lit testing room with a trained healthcare nurse who conducted the healthcare assessment. Any participant who normally wore glasses or hearing aids did so during the task. Participants were seated approximately 60 cm from a computer (Dell Latitude E6400 with Intel Core 2 Duo CPU, 2 Gb RAM, using Windows 7 Professional OS, 60 Hz refresh rate) and instructed to fixate on a fixation cross located at the centre of the screen. This fixation cross (1000 ms) signalled the start of each trial. A visual stimulus (a white disc, 1.5° visual angle, approximately 32 fl luminance) and/or auditory (brief bursts 3500 Hz sounds (10 ms, 1 ms ramp)) stimuli were subsequently presented. The visual stimulus was presented on a black background, positioned 5 cm beneath the of central fixation cross (approximately 4.7° visual angle), for 16 ms. The auditory stimuli were presented at approximately 80 dB over the computer speakers.

The main testing block contained multisensory illusory trials (2B1F, in which B is beep and F is flash), non-illusory trials (2B2F, 1B1F) and unisensory visual trials (0B2F, 0B1F), each presented twice and in a random order across participants. The participants reported the number of perceived visual flashes. Unisensory auditory trials (2B0F) were presented in a separate block and participants reported the number of perceived auditory beeps. Participants’ vocal responses were recorded by the nurse who pressed the corresponding number key on a laptop. The next trial was commenced when the nurse pressed the space bar. Illusion trials of the SIFI consisted of three SOAs, 70 ms, 150 ms and 230 ms, and the second beep either preceded (pre) or followed (post) the flash-beep pair. Due to time constraints within the overall TILDA protocol, a total of twelve ‘illusory’ trials were completed (i.e., two trials per SOA across pre-post conditions).

### Analysis

Response accuracy to illusion trials (2 beeps with 1 flash) of the SIFI task constituted the main outcome of this study. An accurate response indicated that the participant was not susceptible to the illusion on that trial. As there were only two trials per condition of the SIFI within TILDA, accuracy took the form of 0 (i.e., an incorrect response on both trials, indicating illusion susceptibility), 0.5 (correct response on one trial) or 1 (correct response on both trials) and was treated as a discrete variable. We analysed these data with a generalised logistic mixed effects regression model via the ‘lme4’ package^28^ and we controlled for numerous covariates, as in previous studies involving the TILDA cohort^10,11,13^. The following terms were included in the model: age group (53–59, 60–69, 70 + years), stimulus onset asynchrony (SOA; 70, 150, 230 ms), fall number trajectory group, TUG trajectory group, sex (male, female), education (primary, secondary, tertiary), self-reported vision and audition (poor to excellent), visual acuity score (VAS = 100 − 50 × LogMAR), hearing aid use (yes, no), number of cardiac (0, 1, 2 +) and non-cardiac (0, 1, 2 +) diseases, number of emotional/nervous/psychiatric problems (0, 1, 2 +), body mass index (BMI; kg/m^2^), MoCA score, accuracy (0, 0.5, 1) on multisensory congruent (1 beep and 1 flash), unimodal visual (2 flashes only; 70 ms SOA) and unimodal auditory trials (2 beeps only; 70 ms SOA) and pre-post condition. The analysis was conducted with R^29^ in R studio^30^. Supplementary materials (anchored K-medoids criterion plot, full model results and R script) are available at https://osf.io/uxgdj/?view_only=e1c290a12fcf40abbd2806a5f2408c89. In addition to the fixed effects and covariates described previously, the analysis model included age*SOA, sex*SOA, MoCA*SOA and pre-post*SOA interaction terms and a random intercept term for participant ID. All continuous variables were scaled prior to analysis.

## Results

### Number of falls and fall risk trajectories

Longitudinal anchored k-medoids clustering suggested a solution of three trajectory groups for both fall number and sensorimotor fall risk. This was verified with visual inspection of Calinski-Harabasz^31^ criterion plots, a well-established method of determining the optimal clustering solution (see Supplementary Materials). Both solutions of three trajectory groups were considered clinically meaningful and therefore appropriate for addressing our research question.

**Figure 2:**
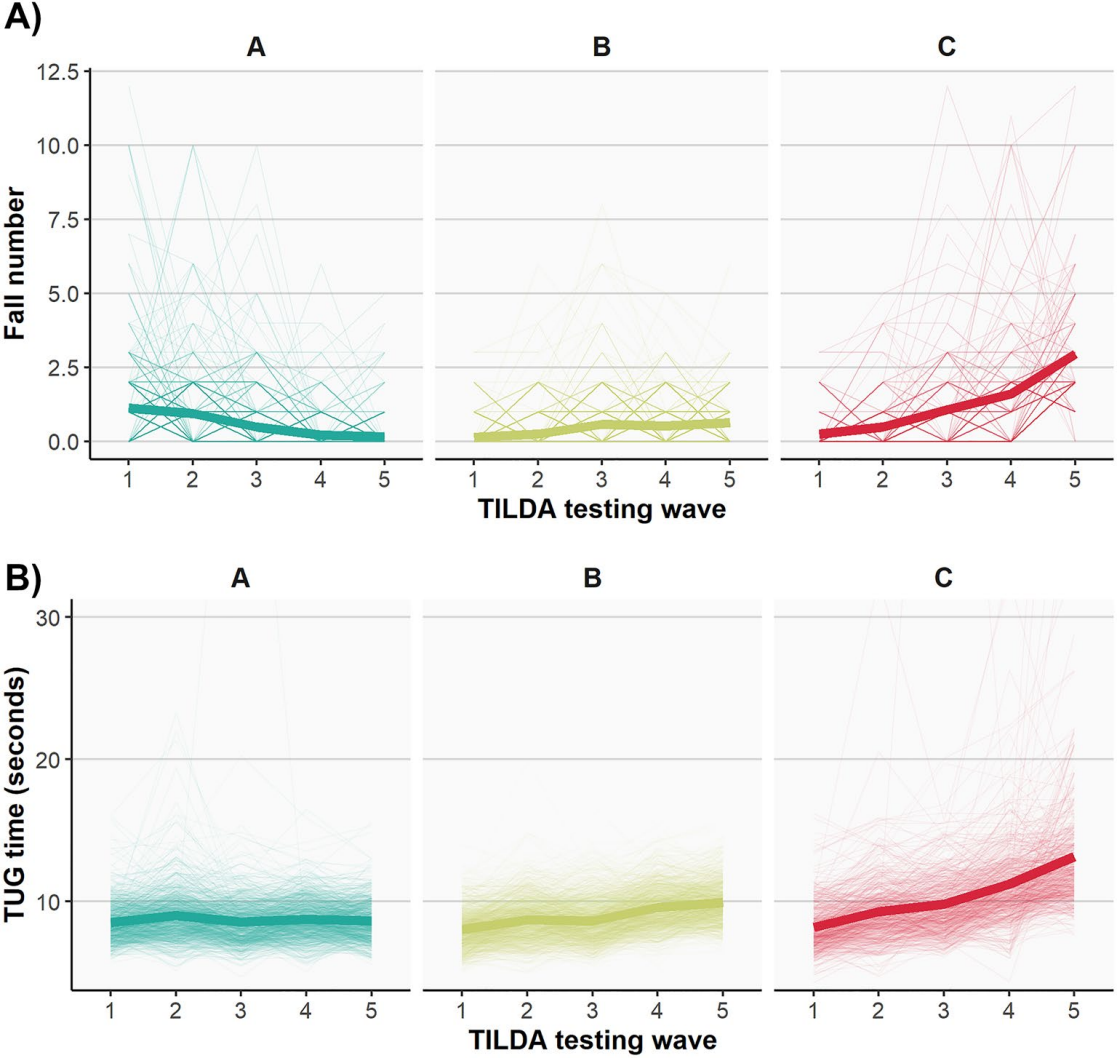
Longitudinal trajectories derived from anchored k-medoids clustering using data from testing waves 1 to 5. (**A**) Fall number trajectories, based on self-reported fall number, where higher values represent more falls reported. A = Decreasing fall number trajectory. B = Stable fall number trajectory. C = Increasing fall number trajectory. (**B**) Sensorimotor fall risk trajectories based on Timed Up and Go performance, where higher values indicate slower TUG suggesting reduced mobility and higher fall risk. A = Low fall risk. B** = **Moderate fall risk. C** = **High fall risk. All plots show the group mean longitudinal trajectories (thick lines) and individual trajectories (thin lines).

The three trajectories of fall numbers consisted of those with a decreasing (*n* = 514), a relatively stable (*n* = 631) or an increasing (*n* = 171) number of falls over 10 years as shown in Fig. 2A. Pairwise Wilcoxon signed rank tests confirmed that the median number of falls at wave 1 was significantly greater than that at wave 5 for the decreasing fall number trajectory (1 vs. 0*; p* < 0.001, *r* = 0.76) and significantly fewer for both the stable fall number (0 vs. 1; *p* < 0.001, *r* = 0.64) and increasing fall number (0 vs. 2; *p* < 0.001, *r* = 0.87) trajectories. As non-fallers were not included in the clustering approach but constituted a distinct, standalone group, there were four groups for fall number in total which were included in our analysis (non-fallers, decreasing fallers, stable fallers and increasing fallers).

For the measure of fall risk, the three trajectories consisted of those with a relatively stable sensorimotor TUG performance (*n* = 847; low fall risk), moderately declining TUG performance (i.e., slower over waves; *n* = 934; moderate fall risk) or more severely declining TUG performance (*n* = 538; high fall risk) over 10 years as shown in Fig. 2B. Pairwise t-tests confirmed that the low fall risk trajectory group showed a significant decrease (i.e., faster) in mean TUG time across 10 years (*Mean*_*diff*_ =  + 0.11 s, *SD* = 1.02 *p* = 0.001, *d* = 0.11), whereas a significant increase (i.e., slower) in mean TUG time was observed for the moderate fall risk (*Mean*_*diff*_ =  + 1.90 s, *SD* = 0.83; *p* < 0.001, *d* = 2.28) and high fall risk (*Mean*_*diff*_ =  + 4.97 s, *SD* = 5.09; *p* < 0.001, *d* = 0.98) trajectories. Further group descriptives are available in Supplementary Materials, Tables S1, S2 and S3.

### Multisensory integration (SIFI performance)

To address our hypothesis that number of falls and/or fall risk would be associated with the precision of temporal multisensory integration in older adults, we examined whether the effect of SOA on accuracy, using the 70 ms as the reference condition, interacted with age group and either fall number or TUG trajectory group. This enabled us to answer whether the pattern of multisensory integration across time was dependant on age and the nature of the fall number or fall risk trajectory. We included an interaction with age group as the results of previous studies suggest that the relationship between falling and overall SIFI susceptibility may be due to differences in age across the different cohorts tested^19^. We opted to treat age as a discrete as opposed to continuous variable to minimise the potential for an outsized influence of a small number of older adults on either end of the age range on the results and to ease the interpretation of a complex, three-way interaction. Moreover, this approach is in line with previous work from our group involving the TILDA cohort in which the effect of age on SIFI susceptibility was assessed^10^. The statistical significance of the AgeGroup*SOA*FallGroup interaction term and the AgeGroup*SOA*TUGGroup interaction term was determined by likelihood ratio tests: that is, we compared the fit of a model with and without each interaction term independently while holding all other terms constant. As we conducted two likelihood ratio tests, the alpha level was adjusted using the Bonferroni correction to adjust for these multiple comparisons (corrected α = 0.025). For fall history, the number of falls reported by non-fallers served as the reference group. For sensorimotor fall risk, performance of the trajectory group with the most stable TUG performance (i.e., the lowest fall risk) served as the reference group.

**Figure 3:**
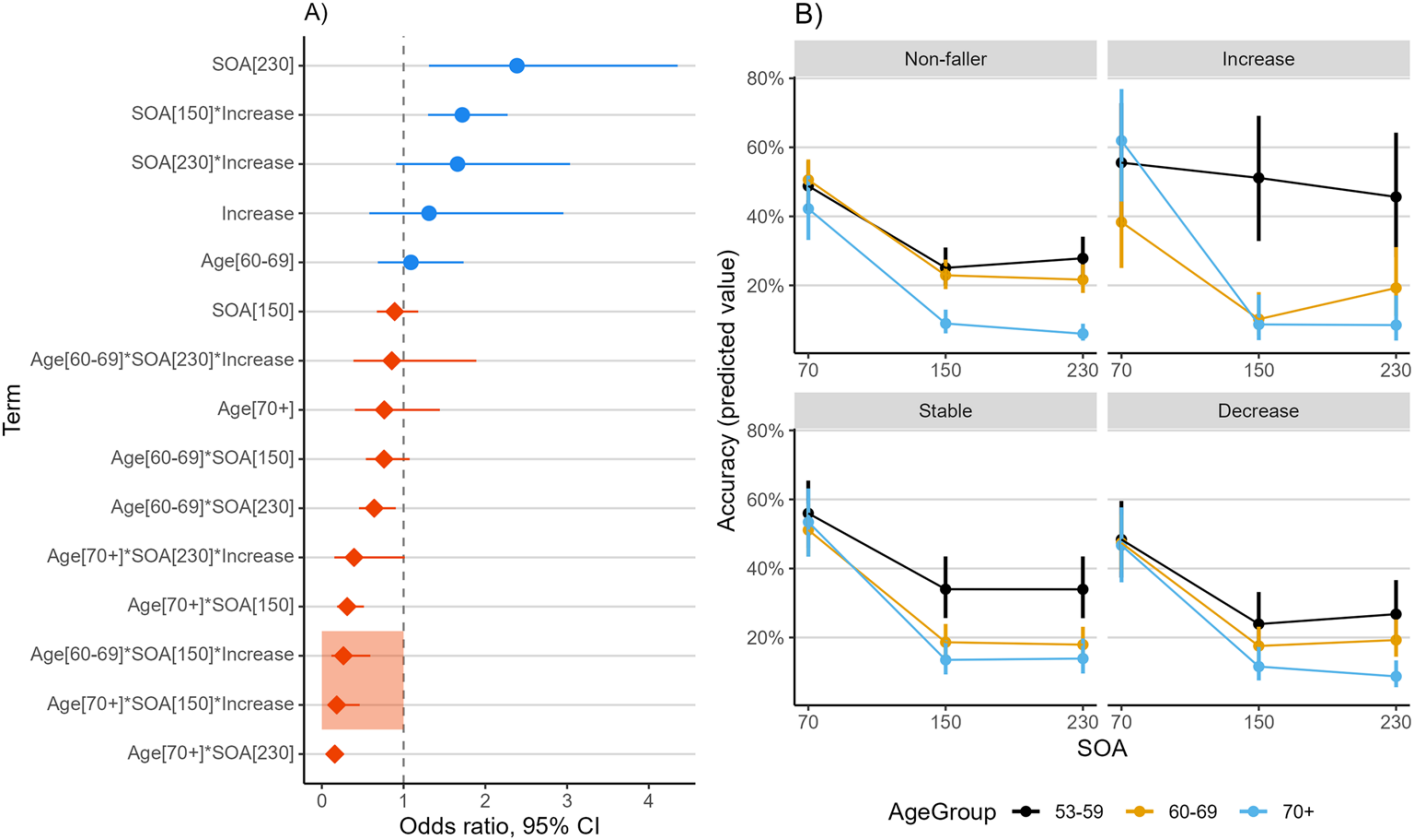
Plots show a summary of the analysis results of a generalised logistic mixed-effect regression model predicting accuracy on the illusory (i.e., 2B1F) trials of the SIFI task. (**A**) The plot shows the odds ratios for key model terms with 95% confidence intervals. Odds ratios to the left of the dashed line (red diamonds) signify a lower odds of making an accurate response (i.e., increased illusion susceptibility) and odds ratios to the right of the dashed line (blue circles) signify increased odds of making an accurate response (i.e., reduced illusion susceptibility). (**B**) The predicted accuracy values (marginal means) across levels of age group and SOA of the SIFI trials for the increasing fall number trajectory group versus the non-fallers.

For fall number, the likelihood ratio test revealed that the AgeGroup*SOA*FallGroup term significantly contributed to the model predicting accuracy on illusion trials of the SIFI (χ^2^_(12)_ = 32.80, *p* = 0.001) as shown in Fig. 3A. This interaction was driven by age-related differences in SIFI susceptibility between the SOAs of 70 ms and 150 ms for the increasing fall trajectory group versus the non-fallers (the contrast in performance accuracy at 230 ms versus 70 ms across age and fall trajectory groups did not reach significance). More specifically, relative to the non-fallers, older adults with an increasing fall trajectory aged between 60 and 69 years had a 74% (approximately) lower odds of making an accurate response at 150 ms (odds ratio = 0.26, 95% CI [0.12,0.59], *p* = 0.001) than 70 ms compared to the 53–59 year olds from the same increasing fall trajectory group. As shown in Fig. 3B, this was driven by the 53–59 year old increasing fallers who had comparable illusion susceptibility at both 70 ms and 150 ms while those aged 60–69 years olds showed greater illusion susceptibility at 150 ms (similar to the non-fallers). While the difference in predicted accuracy values (marginal means) between 70 and 150 ms for the 53–59 year olds versus the 60–69 year olds in the non-faller group was 4%, this difference rose to 23% for the those in the increasing number of falls trajectory group (see Table 1).

**Table 1:** Predicted accuracy values [marginal means; 95% CIs] of a correct response for each fall number trajectory group at 70 ms and 150 ms SOAs of the SIFI task across age groups.

Age Group (*M* ± *SD*)	Non-fallers			Age Group *(M* ± *SD)*	Increase		
	70	150	70 v 150		70	150	70 v 150
**53–59** (56.5 ± 1.60)	49% [42,56]	25% [20,31]	24%	**53–59** (56.6 ± 1.64)	56% [37,73]	51% [33,69]	5%
**60–69** (64.2 ± 2.91)	51% [45,56]	23% [19,27]	28%	**60–69** (64.8 ± 3.02)	38% 25,54	10% [6,18]	28%
**70 + **(74.4 ± 3.89)	42% [33,52]	9% [6,13]	33%	**70 + **(74.7 ± 4.21)	62% [44,77]	9% [4,17]	53%

	Stable				Decrease		
	70	150	70 v 150		70	150	70 v 150
**53–59** (56.7 ± 1.63)	56% [46,65]	34% [26,43]	22%	**53–59** (56.8 ± 1.64)	48% [37,60]	24% [17,33]	24%
**60–69** (64.3 ± 2.92)	51% [43,59]	19% [14,24]	32%	**60–69** (64.3 ± 2.71)	47% [39,56]	18% [13,23]	29%
**70 + **(74.8 ± 4.22)	53% [43,63]	14% [9,19]	39%	**70 + **(74.6 ± 4.01)	47% [36,58]	12% [8,17]	35%

Lower percentages indicate higher illusion susceptibility. A larger positive difference between 70 and 150 ms indicates greater susceptibility in the latter compared to the former condition.

Compared to the non-fallers, older adults in the increasing number of falls trajectory group aged 70 + years had an 82% (approximately) lower odds of making an accurate response at 150 ms (odds ratio = 0.18, 95% CI [0.07,0.46], *p* < 0.001) than to 70 ms compared to the 53–59 year olds. As shown in Fig. 3B, this was driven not only by the 53–59 year old increasing fallers maintaining their performance at 150 ms relative to 70 ms but also by the large difference in accuracy between 70 and 150 ms by the 70 + year old increasing fallers. Indeed, while the difference in predicted accuracy values (marginal means) between 70 and 150 ms for the 53–59 year olds versus the 70 + year olds in the non-faller group was 9%, this difference was 48% for the increasing number of falls trajectory group (see Table 1).

In contrast to the results based on number of falls, the analyses of sensorimotor fall risk, measured by TUG, based on the likelihood ratio test revealed that the Age*SOA*TUGGroup term did not significantly contribute to the model predicting accuracy on the illusion trials of the SIFI (χ^2^_(8)_ = 8.50, *p* = 0.39). Full models results are available in Supplementary Materials (Table S6).

## Discussion

We investigated the relationship between longitudinal trajectories of fall incidents, based on self-reported fall number, and sensorimotor fall risk, based on Timed Up and Go (TUG) performance, and multisensory integration in a sample of 2319 community-dwelling older adults. In line with previous studies of multisensory processing, both within the TILDA cohort^10,11,13^ and from the results of other studies [e.g.,^4,5,14,32^], older adults were more susceptible to the Sound Induced Flash Illusion (SIFI) at long SOAs compared to the shortest SOA, indicating reduced precision in temporal multisensory integration. Our findings extend previous work by demonstrating that the association between age and SOA is further influenced by longitudinal trajectories capturing fall number but not sensorimotor fall risk over 10 years. This finding is consistent with previous evidence suggesting links between falling and distinct patterns of multisensory integration in ageing^14,15,17,18^.

However, in our current study, higher overall SIFI susceptibility (i.e., collapsing across SOA) was not exhibited by the older adult fallers; moreover, the effect observed was specific to increasing fallers rather than fallers more broadly and was modulated by age. This is dissimilar to previous findings^14,15^, likely due to fundamental differences in the sample size, sample characteristics, methodology and/or approach to analysis between these studies. Furthermore, neither the stable nor decreasing fall trajectory groups significantly differed in SIFI task performance compared to the non-fallers. This finding suggests that, in the TILDA cohort, a specific pattern of falling over time is associated with the precision of temporal multisensory integration rather than the act of falling more generally. The group of older adults with an increasing trajectory of incidents of falls may be more likely to represent intrinsic, pathological issues such as perturbed balance function, which may in turn be specifically associated with multisensory integration^16^. Indeed, there is evidence for distinct physical and cognitive profiles of recurrent (versus non-recurrent) fallers^33,34^: the highest percentage of older adult fallers reporting unexplained falls over 10 years was observed in the increasing fall trajectory group (see Supplementary Materials, Table S1) and *all* were recurrent fallers by the end of the 10-year period (see Supplementary Materials, Table S1). Moreover, a higher percentage of older adults in this group reported unsteadiness while walking, standing and transitioning from a seated to standing position compared to the other trajectory groups (see Supplementary Materials, Table S1).

Typically, older adults show increased susceptibility to the SIFI at long compared to short SOAs, which may be, to an extent, a hallmark of ‘healthy’ ageing^4,5,10^. In contrast, the 53–59 year old increasing fallers here uniquely showed equivalent levels of SIFI susceptibility at both SOAs of 70 ms and 150 ms (accuracy was also well maintained at the longest SOA of 230 ms for this group, as shown in Fig. 3B). In this respect, these 53–59 year old recurrent fallers are not showing the same pattern of SIFI susceptibility as either healthy young adults reported from previous studies^5–7^, or their aged-matched non-faller counterparts who are more susceptible at longer SOAs. The reasons for this finding are unclear, as the few studies examining SIFI in the context of falling have typically involved adult fallers who are much older than the age of 53–59 years [see e.g.,^14,15,19,20^]. Our models controlled for self-reported vision and hearing ability, hearing aid use, visual acuity and unimodal visual and auditory accuracy (at 70 ms), which indicates that any group and age differences in sensory function cannot fully explain the reduced illusion susceptibility in these fallers. One possibility is that there is stronger visual upweighting in the 53–59 year olds with increasing fall trajectories which helped maintain their accuracy to the SIFI trials over longer audio-visual asynchronies. By fifty years of age, a decline in balance and postural control is already underway^35,36^ and some reports suggest an increased reliance on visual information to compensate for noisier and less reliable vestibular and proprioceptive function^35–37^. However, this amplified visual weighting may result in challenges to postural control in situations where visual signals are inconsistent, conflicting or unreliable (e.g., in low light or visually dynamic environments), potentially leading to a fall. Compared to non-fallers, older adult fallers may exhibit increased visual field dependence^38,39^, meaning that they strongly rely on visual input for balance/postural control and navigation. As such, strong visual upweighting due to a reliance on visual context, to compensate for reduced balance function, could result in sustained accuracy on the SIFI trials across SOAs. As all participants were seated during the SIFI task, this proposal might also mean that increasing fallers aged 53–59 years upweight vision even in multisensory contexts where their balance and posture are not actively challenged; however, visual field dependence may be a persistent perceptual characteristic^38–40^, particularly evident in fallers with a high risk of future falls as well as a history of recurrent falls^38,39^, consistent with the increasing fall trajectory group. Importantly, there is evidence that SIFI susceptibility can be influenced by the reliability of the sensory information (visual and auditory) presented during the illusion^41,42^. We recognise that although this proposal is, at present, speculative it is one that is amenable to testing in future empirical studies.

In contrast to the 53–59 year old recurrent fallers, the 70 + year old recurrent fallers showed increased illusion susceptibility at 150 ms (as well as 230 ms) while maintaining relatively high accuracy at an SOA of 70 ms. A parsimonious explanation for reduced SIFI susceptibility at 70 ms with sustained susceptibility at longer SOAs is a reduced ability to perceive two beeps presented close together in time. However, as mentioned above, our model controlled for accuracy in detecting two beeps alone [see also^43,44^]. Instead, it may be pertinent to consider differences in task instructions between the unimodal auditory and illusory conditions. That is, participants in the TILDA study were explicitly instructed to indicate how many beeps they heard during the unimodal auditory trials only but *not* during the bimodal illusion trials of the SIFI. Presumably, these instructions encouraged the allocation of selective attention to the beeps in the unimodal condition. Selective attention can enhance auditory processing through the amplification and sharpening of neural responses via mechanisms such as inhibition^45,46^. This may be particularly important for older adults who, because of age-related changes in the periphery, may process increased sensory noise compared to their younger counterparts^47^ and show less precise temporal processing^48–50^. However, there is evidence for reduced top-down inhibitory control of auditory processing from prefrontal cortex in ageing^51,52^. Inhibitory control is linked with the perception of temporal events^53–55^ as well as outcomes such as perturbed gait, balance function and falls^1,22,56–58^. Furthermore, dysregulation of the prefrontal cortex is hypothesised to link patterns of multisensory integration with perturbed mobility in ageing^59^.

Recently, Scurry et al.^15^ reported an increase in gamma band oscillatory power without a simultaneous modulation of alpha band oscillatory power during the SIFI task in older adults with a history of falls (70 + years), but not in healthy older adult (non-fallers) or young adults. They proposed that this finding for older fallers reflected imprecise bottom-up auditory processing (gamma band) without sufficient top-down inhibitory control (alpha band). Scurry et al.^15^ exposed the participants in their study to only a single SOA in the illusion condition; therefore, comparisons with the present study are limited. Nevertheless, reduced inhibitory function in the 70 + year old recurrent fallers may have, in the absence of selective attention, reduced the fidelity of the auditory signals at an SOA of 70 ms. Less precise discrimination of auditory ‘beeps’ may have reduced susceptibility to the SIFI on these trials (indeed, lowering the reliability of the auditory signals has been shown to directly reduce illusion susceptibility^41^). In contrast, increasing the SOA duration to 150 ms presumably facilitated the discrimination of a second beep that was then insufficiently inhibited, accounting for the increase in susceptibility to the SIFI on these trials.

To investigate whether inhibitory function is associated with patterns of susceptibility to the SIFI, we examined participants’ performance on the Sustained Attention to Response Task (SART^60^). This allows us to make some, although limited, assessment of inhibition: SART is a measure of response inhibition in a unimodal context, not perceptual inhibition in a cross-modal context. Details of this analysis are provided in Supplementary Materials. Interestingly, only the increasing fall trajectory group showed a significantly steeper age-related increase in commission errors on the SART relative to the non-fallers (see Supplementary Materials), suggesting a larger age-related decline in inhibitory function for this group. Previous work with the TILDA cohort has provided evidence for associations between poorer SART performance and increased SIFI susceptibility at longer SOAs^11^, suggesting some involvement of inhibitory processes which deserve further investigation.

Collectively, our findings suggest that older adults with an increasing fall trajectory over time may, in the context of the SIFI, change in the precision of their temporal multisensory integration as they get older to a greater extent than older adult non-fallers. We have hypothesised that this change could represent a shift from a benefit of visual upweighting in 53–59 year old recurrent fallers to a detrimental effect of accelerated age-related changes to other bottom-up and/or top-down processes in 70 + year old recurrent fallers. Importantly, these effects on the precision of temporal multisensory integration appear somewhat subtle in the TILDA cohort, as we did not find group differences across age groups at the longest SOA. The specificity of this group difference to older adults with an increasing fall trajectory is potentially clinically meaningful, particularly if less precise multisensory processing is predictive of future falls^17^. In this respect, while the fall status of the older adults in the increasing fall trajectory group was mixed at the time they completed the SIFI test in wave 3 (38% non-fallers, 62% fallers), all of these individuals experienced recurrent falls within the subsequent 5 years following SIFI testing. However, it is important to note that the precision of temporal multisensory integration in ageing is modifiable with perceptual training^32^, or indirectly by improving balance function^20^, opening up a potential novel method for mitigating fall incidents. However, since we cannot interpret directionality or causality at the present time (as SIFI data are currently available at wave 3 only), and the influences from other domains can be complex in nature, some caution is warranted when interpreting any potential clinical application of these findings. Moreover, an additional limitation here is that the fall trajectories were derived from self-reported fall number [as in^14,15,19^] but no formal definition of a fall was given to the participants. As such, future work should incorporate a formal description of a fall to older adult participants [e.g., as in^15^] to ensure that older adults are accurately reporting fall incidents based on a consistent definition. Moreover, we recognise that our findings are specific to the integration of external audio-visual events. Future work may consider investigating if falling influences temporal multisensory integration for sensory inputs more intimately linked to the body itself, such as proprioception and somatosensation, in a similar manner to that reported here. Indeed, there is evidence that the body representation, which is shaped by these sensory inputs, changes with age^61,62^ and that these changes are potentially linked with broader age-related sensorimotor decline^62^. Moreover, distinct patterns of visuo-tactile and visuo-proprioceptive integration are reported to be associated with falls or indices of increased fall risk^17,18,59,63,64^. Incidentally, a visuo-tactile version of the SIFI has been reported^65^ and this could serve as an appropriate test for assessing whether distinct age-related patterns of temporal audio-visual integration in fallers are replicated in other modalities. These findings indicate a link between aspects of physical functioning and the integration of sensory inputs more broadly in ageing.

There was no evidence for a significant moderating effect of fall risk trajectories, based on TUG performance, on the interaction between age group and SOA. The TUG task is a brief, relatively accessible, measure of functional mobility, which is designed to assess gait, muscle strength, balance and executive function^25^. If the link between multisensory integration and fall incidence is driven by suboptimal balance or postural control^14^, as suggested by previous studies^17^, the TUG task alone may not be sufficiently sensitive to key aspects of postural maintenance, particularly in a relatively high-functioning cohort [e.g., see^34,66^]. In contrast, a fall is, by definition, a loss of balance or postural control^67^. Given the putative role of cognitive function in the association between fall trajectories and SIFI susceptibility in older adults, an increasing number of falls over time may be more reflective of significant cognitive changes than a slowing of TUG times in this cohort. For example, it has been proposed that inhibitory function is related to falls in older adults^22,58^ but not necessarily to TUG performance [^68^; see also Supplementary Materials] and the longitudinal association between cognition and TUG times in the TILDA cohort are reported to be small in effect size^69^. However, we cannot rule out the possibility that the relationship between long-term mobility and multisensory integration is nuanced, requiring a more extensive range of SOAs and/or a larger trial number than available in our current SIFI paradigm within the TILDA study (indeed, we acknowledge that the necessarily restricted number of trials and SOAs is a limitation of this study more broadly). Nevertheless, the association of longitudinal fall incidents but not general sensorimotor function with SIFI susceptibility is an important and novel finding. It suggests that the precision of temporal audio-visual integration is linked with aspects of physical functioning specific to the experience of falling repeatedly over time and, therefore, that multisensory processing may be a relevant measure for important clinical outcomes. Our findings, arising from a large sample of older adults across a wide age range, can be validated with further lab-based experimental work.

## Conclusions

In conclusion, we investigated the relationship between longitudinal trajectories of fall number and sensorimotor fall risk (based on Timed Up and Go performance) over 10 years on a measure of multisensory integration (via the Sound Induced Flash Illusion; SIFI) in 2319 community-dwelling older adults. The precision by which auditory and visual events were integrated across time was influenced by fall number but not sensorimotor fall risk trajectories. These novel findings shed new light on the complex interplay between falls and multisensory integration in older adults and suggest neural links between key domains in the maintenance of healthy ageing.

## Supplementary Information

Supplementary Information.

## Data Availability

The data from The Irish Longitudinal Study on Ageing (TILDA) is available upon request. Data from the Sound Induced Flash Illusion are available in the publicly released dataset which can be accessed via a hotdesk facility or by requesting access through the Irish Social Science Data Archive or the Interuniversity Consortium for Political and Social Research. Information on how to access these data as well as direct links to the request forms can be found at https://tilda.tcd.ie/data/accessing-data/ and https://tilda.tcd.ie/data/accessing-data/hotdesk/.
