# Supplementary material for: The temporal precision of audiovisual integration is associated with longitudinal fall incidents but not sensorimotor fall risk in older adults
Source: Sci Rep. 2023 May 3;13:7167. doi: 10.1038/s41598-023-32404-y (PMC10156851; doi:10.1038/s41598-023-32404-y)
Supplement: Supplementary file 1 — Supplementary Information. [file 41598_2023_32404_MOESM1_ESM.docx]

**Supplementary Materials**

**The temporal precision of audiovisual integration is associated with longitudinal fall incidents but not** **sensorimotor fall risk in older adults**

**Alan O’ Dowd^*^; Rebecca J. Hirst; Annalisa Setti; Orna A. Donoghue; Rose Anne Kenny; Fiona N. Newell.**

| **Table S1.**  Descriptive statistics for numerical (mean, SD) and categorical (count, %) variables per fall number trajectory group (non-fallers, decreasing, moderately increasing and severely increasing) at wave 3, when multisensory integration was assessed. Overall significant group effects are indicated by bold *p* values across variables. | | | | | |
| --- | --- | --- | --- | --- | --- |
|  | **Nonfaller**  **(n = 1,003)** | **Decrease**  **(n = 514)** | **Stable**  **(n = 631)** | **Increase**  **(n = 171)** | ***p*** |
| **Mean (SD)** |  |  |  |  |  |
| Age, yrs | 63.5 (6.8) | 65.2 (7.2) | 65.3 (7.5) | 65.8 (7.7) | **< .001** |
| VAS | 96.8 (8.3) | 96 (9.3) | 96.5 (8.4) | 95.8 (7.8) | .26 |
| TUG, s | 8.7 (1.6) | 8.9 (3.3) | 9 (2.6) | 9.5 (2.1) | **<.001** |
| Gait speed, cm/s^a^ | 140 (17.1) | 137 (20) | 136 (17.8) | 130 (21.1) | **< .001** |
| Grip strength, kg^b^ | 30 (9.4) | 26.3 (8.6) | 26 (8.8) | 25.6 (9.4) | **< .001** |
| MoCA score | 26.9 (2) | 26.9 (2) | 27.1 (2) | 26.9 (2) | .36 |
| **Count (%)** |  |  |  |  |  |
| Participant sex (female) | 453 (45%) | 307 (60%) | 400 (63%) | 101 (59%) | **<.001** |
| Orthostatic hypotension^c^ | 50 (5%) | 34 (7%) | 41 (7%) | 11 (6%) | .42 |
| Cardiac | 29 (3%) | 26 (5%) | 25 (4%) | 12 (7%) | **.03** |
| Noncardiac | 96 (10%) | 68 (13%) | 72 (11%) | 19 (11%) | .18 |
| Emotional/nervous/psychiatric | 49 (5%) | 33 (6%) | 38 (6%) | 25 (15%) | **<.001** |
| Polypharmacy | 126 (13%) | 90 (18%) | 142 (23%) | 55 (32%) | **<.001** |
| Fair/poor vision | 48 (5%) | 33 (6%) | 54 (9%) | 22 (13%) | **<.001** |
| Fair/poor hearing | 134 (13%) | 70 (14%) | 90 (14%) | 27 (16%) | .84 |
| 2B0F (1)^d^ | 461 (46%) | 212 (41%) | 272 (43%) | 75 (44%) | .32 |
| 0B2F (1)^d^ | 106 (11%) | 51 (10%) | 76 (12%) | 18 (11%) | .68 |
| Unsteady walking | 93 (9%) | 64 (12%) | 99 (16%) | 52 (30%) | **<.001** |
| Unsteady standing | 63 (6%) | 61 (12%) | 69 (11%) | 36 (21%) | **<.001** |
| Unsteady sitting to standing | 134 (13%) | 109 (21%) | 122 (19%) | 62 (36%) | **<.001** |
| Fear of falling^e^ | 290 (29%) | 275 (54%) | 315 (50%) | 107 (63%) | **<.001** |
| ≥ 2 falls^e^ | / | 276 (54%) | 270 (43%) | 171 (100%) | **<.001** |
| Unexplained falls^e^ | / | 136 (27%) | 168 (27%) | 74 (43%) | **<.001** |
| Injurious falls^e^ | / | 251 (49%) | 353 (56%) | 92 (54%) | .06 |
| ^a^*n* = 32 missing gait speed; ^b^*n* = 204 missing grip strength; ^c^*n* = 337 missing cardiovascular data; ^d^2B0F (1) = correct response on both trials of the unimodal auditory condition. 0B2F (1) = correct response on both trials of the unimodal visual condition. ^e^self-reported at any wave. | | | | | |

| **Table S2.**  Descriptive statistics for numerical (mean, SD) and categorical (count, %) variables per TUG trajectory group (stable, moderately declining, severely declining) at wave 3, when multisensory integration was assessed. Overall significant group effects are indicated by bold *p* values across variables. | | | | |
| --- | --- | --- | --- | --- |
|  | **Decrease**  **(n = 847)** | **Moderate increase**  **(n = 934)** | **Severe increase**  **(n = 538)** | ***p*** |
| **Mean (SD)** |  |  |  |  |
| Age, yrs | 63 (6.4) | 64.2 (6.8) | 67.6 (8.2) | **<.001** |
| VAS | 97 (8.4) | 96.4 (8.6) | 95.8 (8.5) | **.04** |
| TUG, s | 8.6 (2.2) | 8.7 (1.5) | 9.8 (3.4) | **<.001** |
| Gait speed, cm/s^a^ | 142 (17.5) | 139 (17.4) | 129 (19.2) | **< .001** |
| Grip strength, kg^b^ | 29 (9.7) | 27.8 (8.9) | 25.9 (9) | **< .001** |
| MoCA score | 27 (2) | 27 (2) | 26.7 (2) | **.01** |
| **Count (%)** |  |  |  |  |
| Sex (female) | 454 (54%) | 506 (54%) | 301 (56%) | .69 |
| Orthostatic hypotension^c^ | 48 (6%) | 57 (6%) | 31 (6%) | .84 |
| Cardiac | 26 (3%) | 36 (4%) | 30 (6%) | .06 |
| Noncardiac | 87 (10%) | 101 (11%) | 67 (12%) | .44 |
| Emotional/nervous/psychiatric | 42 (5%) | 64 (7%) | 39 (7%) | .14 |
| Polypharmacy | 99 (12%) | 168 (18%) | 146 (27%) | **<.001** |
| Fair/poor vision | 48 (6%) | 73 (8%) | 36 (7%) | .20 |
| Fair/poor hearing | 97 (11%) | 132 (14%) | 92 (17%) | **.01** |
| 2B0F (1)^d^ | 382 (45%) | 411 (44%) | 227 (42%) | .57 |
| 0B2F (1)^d^ | 98 (12%) | 89 (10%) | 64 (12%) | .25 |
| Unsteady walking | 78 (9%) | 90 (10%) | 140 (26%) | **<.001** |
| Unsteady standing | 67 (8%) | 57 (6%) | 105 (20%) | **<.001** |
| Unsteady sitting to standing | 119 (14%) | 146 (16%) | 162 (30%) | **<.001** |
| Fear of falling^e^ | 317 (37%) | 379 (41%) | 291 (54%) | **<.001** |
| Faller^e^ | 442 (52%) | 527 (56%) | 347 (64%) | **<.001** |
|  | **Fallers (*n* = 442)** | **Fallers (*n* = 527)** | **Fallers (*n* = 347)** |  |
| ≥ 2 falls^e^ | 235 (53%) | 285 (54%) | 197 (57%) | .58 |
| Unexplained falls^e^ | 114 (26%) | 151 (29%) | 113 (33%) | .11 |
| Injurious falls^e^ | 239 (54%) | 270 (51%) | 187 (54%) | .62 |
| ^a^*n* = 32 missing gait speed; ^b^*n* = 204 missing grip strength; ^c^*n* = 337 missing cardiovascular data; ^d^2B0F (1) = correct response on both trials of the unimodal auditory condition. 0B2F (1) = correct response on both trials of the unimodal visual condition. ^e^self-reported at any wave | | | | |


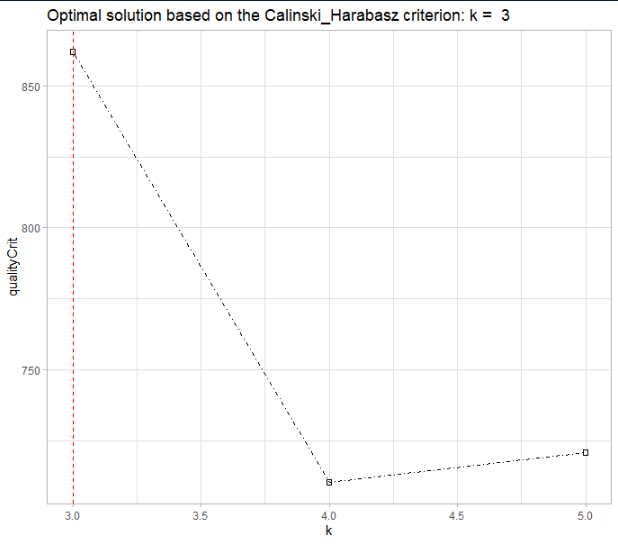

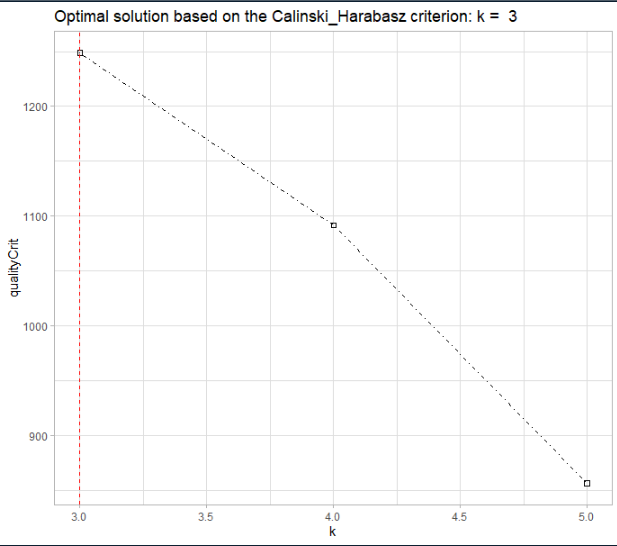


**Figure S1.** [LEFT] Calinski-Harabasz plot for ten-year fall number trajectories, based on Timed Up and Go (TUG) performance. [RIGHT] Calinski-Harabasz plot for ten-year fall number trajectories. Both plots illustrate that the optimal number of trajectories (k) = 3 which has the highest Calinski-Harabasz value, as indexed by the dashed red line.

| **Table S3.**  Counts and percentages of participants in each fall number trajectory group who were organised into each TUG trajectory group | | | |
| --- | --- | --- | --- |
|  | **TUG trajectory group** | | |
|  | **Decrease** | **Moderate increase** | **Severe increase** |
| **Fall number trajectory group** |  |  |  |
| **Non-faller** | 405 (40%) | 407 (41%) | 191 (19%) |
| **Decrease** | 202 (39%) | 196 (38%) | 116 (23%) |
| **Stable** | 192 (30%) | 262 (42%) | 177 (28%) |
| **Increase** | 48 (28%) | 69 (40%) | 54 (32%) |

| **Table S4.** Mean accuracy (SD) for the fall number trajectory groups at 70 ms, 150 ms and 230 ms SOAs of the SIFI task across age groups. | | | | | | | |  |
| --- | --- | --- | --- | --- | --- | --- | --- | --- |
| **AgeGroup** (*M*±*SD*) | **Non-fallers** | | | **AgeGroup** *(M*±*SD)* | **Increase** | | |  |
|  | **70** | **150** | **230** |  | **70** | **150** | **230** |  |
| **53-59**  (56.5±1.60) | 0.49 (0.40) | 0.36 (0.37) | 0.39 (0.39) | **53-59**  (56.6±1.64) | 0.57 (0.42) | 0.54 (0.40) | 0.51 (0.39) |  |
| **60-69**  (64.2±2.91) | 0.50 (0.41) | 0.35 (0.38) | 0.35 (0.37) | **60-69**  (64.8±3.02) | 0.43 (0.40) | 0.23 (0.34) | 0.31 (0.36) |  |
| **70+**  (74.4±3.89) | 0.45 (0.41) | 0.21 (0.32) | 0.18 (0.28) | **70+**  (74.7±4.21) | 0.55 (0.40) | 0.21 (0.37) | 0.21 (0.33) |  |
|  | **Stable** | | |  | **Decrease** | | |  |
|  | **70** | **150** | **230** |  | **70** | **150** | **230** |  |
| **53-59**  (56.7±1.63) | 0.57 (0.39) | 0.44 (0.40) | 0.44 (0.39) | **53-59**  (56.8±1.64) | 0.52 (0.41) | 0.37 (0.39) | 0.38 (0.38) |  |
| **60-69**  (64.3±2.92) | 0.51 (0.40) | 0.29 (0.35) | 0.29 (0.35) | **60-69**  (64.3±2.71) | 0.51 (0.41) | 0.30 (0.37) | 0.32 (0.38) |  |
| **70+**  (74.8±4.22) | 0.52 (0.41) | 0.26 (0.35) | 0.26 (0.35) | **70+**  (74.6±4.01) | 0.48 (0.40) | 0.25 (0.34) | 0.21 (0.31) |  |

| **Table S5.** Predicted values [marginal means; 95% CIs] of a correct response for each fall number trajectory group at 70 ms, 150 ms and 230 ms SOAs of the SIFI task across age groups. Lower percentages indicate higher illusion susceptibility. | | | | | | | |  |
| --- | --- | --- | --- | --- | --- | --- | --- | --- |
| **AgeGroup** (*M*±*SD*) | **Non-fallers** | | | **AgeGroup** *(M*±*SD)* | **Increase** | | |  |
|  | **70** | **150** | **230** |  | **70** | **150** | **230** |  |
| **53-59**  (56.5±1.60) | 49%  [42,56] | 25% [20,31] | 28% [22,34] | **53-59**  (56.6±1.64) | 56%  [37,73] | 51%  [33,69] | 46%  [28,64] |  |
| **60-69**  (64.2±2.91) | 51%  [45,56] | 23% [19,27] | 22%  [18,26] | **60-69**  (64.8±3.02) | 38%  [25,54] | 10%  [6,18] | 19% [11,31] |  |
| **70+**  (74.4±3.89) | 42 %  [33,52] | 9%  [6,13] | 6%  [4,9] | **70+**  (74.7±4.21) | 62%  [44,77] | 9%  [4,17] | 9%  [4,17] |  |
|  | **Stable** | | |  | **Decrease** | | |  |
|  | **70** | **150** | **230** |  | **70** | **150** | **230** |  |
| **53-59**  (56.7±1.63) | 56%  [46,65] | 34% [26,43] | 34%  [26,43] | **53-59**  (56.8±1.64) | 48%  [37,60] | 24%  [17,33] | 27%  [19,37] |  |
| **60-69**  (64.3±2.92) | 51%  [43,59] | 19% [14,24] | 18%  [14,23] | **60-69**  (64.3±2.71) | 47%  [39,56] | 18%  [13,23] | 19%  [14,25] |  |
| **70+**  (74.8±4.22) | 53 %  [43,63] | 14%  [9,19] | 14%  [10,20] | **70+**  (74.6±4.01) | 47%  [36,58] | 12%  [8,17] | 9%  [6,13] |  |

**Additional analyses – cognition**

To investigate whether the fall groups differed on performance on specific cognitive tasks, we introduced data from the Sustained Attention to Response Task (SART commission and omission errors), the Choice Response Time Task (CRT cognitive and motor reaction times) and the Colour Trails Task (CTT time 1 and CTT time 2). Further information on these tasks is available elsewhere [1]. Individual linear regression models, one for each cognitive task as the outcome variable, were conducted. The predictor of interest was the age*FallGroup interaction, where age was treated was continuous given the continuous nature of the dependent variable. All models included the following covariates: sex, education, TUGGroup, Visual Acuity Score (VAS), self-report vision, self-report hearing, hearing aid use, body mass index, number of cardiac and non-cardiac conditions, number of psychiatric/nervous/emotional problems, accuracy on the congruent 1B1F condition, unimodal 2B0F condition and unimodal 0B2F condition as well as an age*TUGGroup interaction.

Data from participants with missing or outlying values for each cognitive test were omitted and the final sample sizes across models were as follows: *N* = 2,286 for the SART model, *N* = 2,181 for the CRT model and *N* = 2,256 for the CTT model. The cognitive data were also transformed to correct for substantial deviations from normality prior to analysis. The SART commission errors were best corrected with a square root transformation. The SART omission errors, CRT cognitive and motor response times and the CTT 1 and CTT 2 times were best corrected with a log transformation, as verified with visual inspection of plots.

**Results**

A likelihood ratio test confirmed the significant contribution of the age*FallGroup interaction to the model fit predicting SART commission errors (*F*(3) = 2.95, *p* = .03), as shown in Figure S2A below. This was driven by the increasing fall trajectory group exhibiting a significantly steeper increase in SART commission errors with increasing age compared to the non-fallers (β = .19, *p* = .006), as shown in Figure S2B below. The likelihood ratio tests were non-significant for the SART omission errors (*F*(3) = 1.75, *p* = .16), CRT cognitive response times (*F*(3) = 0.90, *p* = .44), CRT motor response times (*F*(3) = .06, *p* = .98), CTT 1 times (*F*(3) = 1.5, *p* = .20) and CTT 2 times (*F*(3) = 0.63, *p* = .60). There was no evidence for any significant age*TUGGroup interaction in any of the cognitive models. However, those in the severely increasing TUG trajectory group (i.e., high fall risk) showed slower motor response times on the CRT overall (*F*(2) = 4.40, *p* = .01; *β* = .05, *p* = .004).


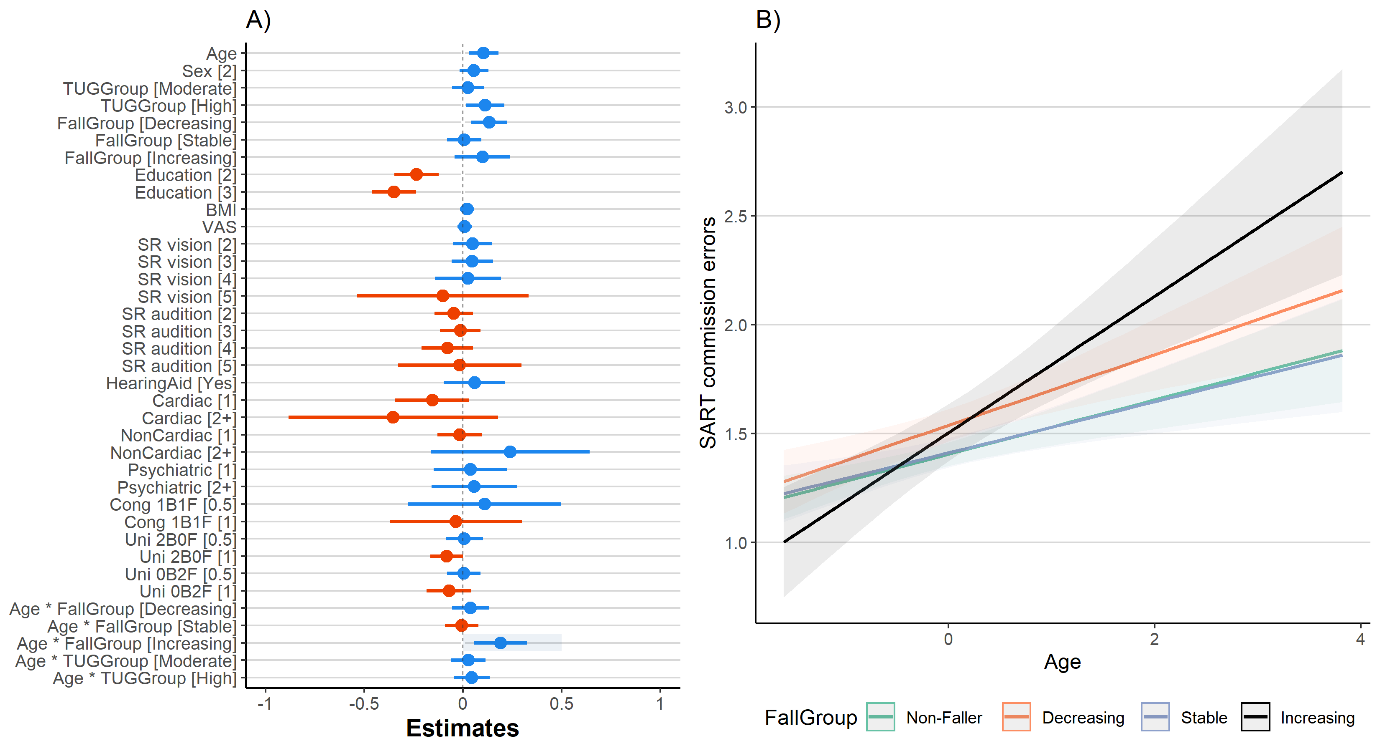


**Figure S2.** Plot shows the results of a linear model predicting SART commission errors. A) Model coefficients. B) Interaction between age and fall number trajectory group.

1. Hirst, R. J., Setti, A., De Looze, C., Kenny, R. A., & Newell, F. N. (2022). Multisensory integration precision is associated with better cognitive performance over time in older adults: A large-scale exploratory study. *Aging Brain*, **2**, 100038 (2022).<https://doi.org/10.1016/j.nbas.2022.100038>

| **Table S6.**  Full results of the main model predicting accuracy on 2B1F trials of the SIFI (Odds ratios) | | | | | |
| --- | --- | --- | --- | --- | --- |
| **Term** | **Odds ratios** | ***p*** | **Lower 95% CI** | **Upper 95% CI** |  |
| (Intercept) | 0.04 | .005 | 0 | 0.38 |  |
| AgeGroup [60-69] | 1.09 | 0.72 | 0.68 | 1.73 |  |
| AgeGroup [70+] | 0.76 | 0.41 | 0.4 | 1.44 |  |
| SOA [150] | 0.89 | 0.42 | 0.67 | 1.18 |  |
| SOA [230] | 1.72 | <.001 | 1.3 | 2.27 |  |
| Sex [Female] | 2.07 | <.001 | 1.66 | 2.59 |  |
| PrePost [Pre] | 0.8 | <.001 | 0.72 | 0.89 |  |
| FallGroup [Decrease] | 0.98 | 0.95 | 0.58 | 1.67 |  |
| FallGroup [Stable] | 1.33 | 0.25 | 0.82 | 2.17 |  |
| FallGroup [Increase] | 1.31 | 0.51 | 0.58 | 2.95 |  |
| TUGGroup [Moderate] | 1.01 | 0.97 | 0.66 | 1.55 |  |
| TUGGroup [High] | 0.67 | 0.17 | 0.37 | 1.19 |  |
| Education [Secondary] | 1.17 | 0.32 | 0.85 | 1.62 |  |
| Education [Tertiary] | 1.24 | 0.19 | 0.9 | 1.71 |  |
| MoCA | 1.15 | 0.02 | 1.03 | 1.29 |  |
| BMI | 1.06 | 0.27 | 0.96 | 1.17 |  |
| VAS | 0.96 | 0.49 | 0.87 | 1.07 |  |
| SR_vision [Fair] | 0.11 | <.001 | 0.03 | 0.4 |  |
| SR_vision [Good] | 0.11 | <.001 | 0.03 | 0.36 |  |
| SR_vision [Very Good] | 0.12 | <.001 | 0.03 | 0.39 |  |
| SR_vision [Excellent] | 0.14 | .002 | 0.04 | 0.49 |  |
| SR_audition [Fair] | 0.45 | 0.08 | 0.18 | 1.1 |  |
| SR_audition [Good] | 0.5 | 0.12 | 0.21 | 1.21 |  |
| SR_audition [Very Good] | 0.42 | 0.06 | 0.17 | 1.02 |  |
| SR_audition [Excellent] | 0.41 | 0.06 | 0.17 | 1.02 |  |
| HearingAid [Yes] | 1.7 | 0.02 | 1.1 | 2.64 |  |
| Cardiac [1] | 1.54 | 0.11 | 0.9 | 2.62 |  |
| Cardiac [2+] | 2.18 | 0.31 | 0.48 | 9.85 |  |
| NonCardiac [1] | 1.25 | 0.17 | 0.91 | 1.73 |  |
| NonCardiac [2+] | 1.16 | 0.8 | 0.37 | 3.61 |  |
| Psychiatric [1] | 1.4 | 0.21 | 0.83 | 2.38 |  |
| Psychiatric [2+] | 1.33 | 0.36 | 0.72 | 2.44 |  |
| Cong_1B1F [0.5] | 194.79 | <.001 | 34.06 | 1114.06 |  |
| Cong_1B1F [1] | 433.58 | <.001 | 83.14 | 2261.08 |  |
| Uni_0B2F [0.5] | 0.57 | <.001 | 0.45 | 0.73 |  |
| Uni_0B2F [1] | 0.5 | <.001 | 0.36 | 0.7 |  |
| Uni_2B0F [0.5] | 0.72 | 0.02 | 0.55 | 0.94 |  |
| Uni_2B0F [1] | 0.88 | 0.29 | 0.7 | 1.11 |  |
| AgeGroup [60-69]:SOA [150] | 0.76 | 0.12 | 0.54 | 1.07 |  |
| AgeGroup [70+]:SOA [150] | 0.31 | <.001 | 0.19 | 0.51 |  |
| AgeGroup [60-69]:SOA [230] | 0.64 | 0.01 | 0.45 | 0.9 |  |
| AgeGroup [70+]:SOA [230] | 0.16 | <.001 | 0.09 | 0.27 |  |
| SOA [150]:Sex [Female] | 0.36 | <.001 | 0.3 | 0.42 |  |
| SOA [230]:Sex [Female] | 0.24 | <.001 | 0.2 | 0.29 |  |
| SOA [150]:PrePost [Pre] | 0.57 | <.001 | 0.48 | 0.67 |  |
| SOA [230]:PrePost [Pre] | 0.39 | <.001 | 0.33 | 0.45 |  |
| SOA [150]:MoCA | 1.31 | <.001 | 1.21 | 1.43 |  |
| SOA [230]:MoCA | 1.44 | <.001 | 1.32 | 1.57 |  |
| SOA [150]:FallGroup [Decrease] | 0.95 | 0.82 | 0.64 | 1.42 |  |
| SOA [230]:FallGroup [Decrease] | 0.96 | 0.85 | 0.65 | 1.43 |  |
| SOA [150]:FallGroup [Stable] | 1.15 | 0.44 | 0.8 | 1.66 |  |
| SOA [230]:FallGroup [Stable] | 1 | 1 | 0.69 | 1.44 |  |
| SOA [150]:FallGroup [Increase] | 2.39 | .005 | 1.31 | 4.35 |  |
| SOA [230]:FallGroup [Increase] | 1.66 | 0.1 | 0.91 | 3.04 |  |
| AgeGroup [60-69]:FallGroup [Decrease] | 0.9 | 0.75 | 0.46 | 1.74 |  |
| AgeGroup [70+]:FallGroup [Decrease] | 1.22 | 0.62 | 0.56 | 2.66 |  |
| AgeGroup [60-69]:FallGroup [Stable] | 0.77 | 0.4 | 0.41 | 1.43 |  |
| AgeGroup [70+]:FallGroup [Stable] | 1.18 | 0.66 | 0.57 | 2.44 |  |
| AgeGroup [60-69]:FallGroup [Increase] | 0.46 | 0.15 | 0.16 | 1.32 |  |
| AgeGroup [70+]:FallGroup [Increase] | 1.7 | 0.36 | 0.54 | 5.32 |  |
| SOA [150]:TUGGroup [Moderate] | 0.9 | 0.52 | 0.65 | 1.24 |  |
| SOA [230]:TUGGroup [Moderate] | 0.69 | 0.02 | 0.5 | 0.95 |  |
| SOA [150]:TUGGroup [High] | 0.83 | 0.4 | 0.53 | 1.28 |  |
| SOA [230]:TUGGroup [High] | 0.82 | 0.38 | 0.53 | 1.27 |  |
| AgeGroup [60-69]:TUGGroup [Moderate] | 0.92 | 0.76 | 0.53 | 1.6 |  |
| AgeGroup [70+]:TUGGroup [Moderate] | 0.83 | 0.61 | 0.41 | 1.69 |  |
| AgeGroup [60-69]:TUGGroup [High] | 1.09 | 0.82 | 0.53 | 2.26 |  |
| AgeGroup [70+]:TUGGroup [High] | 1.4 | 0.42 | 0.62 | 3.15 |  |
| AgeGroup [60-69]:SOA [150]:FallGroup [Decrease] | 0.85 | 0.53 | 0.51 | 1.41 |  |
| AgeGroup [70+]:SOA [150]:FallGroup [Decrease] | 1.16 | 0.63 | 0.63 | 2.15 |  |
| AgeGroup [60-69]:SOA [230]:FallGroup [Decrease] | 1.02 | 0.95 | 0.61 | 1.69 |  |
| AgeGroup [70+]:SOA [230]:FallGroup [Decrease] | 1.3 | 0.42 | 0.69 | 2.44 |  |
| AgeGroup [60-69]:SOA [150]:FallGroup [Stable] | 0.65 | 0.07 | 0.41 | 1.04 |  |
| AgeGroup [70+]:SOA [150]:FallGroup [Stable] | 0.87 | 0.65 | 0.49 | 1.55 |  |
| AgeGroup [60-69]:SOA [230]:FallGroup [Stable] | 0.77 | 0.28 | 0.48 | 1.24 |  |
| AgeGroup [70+]:SOA [230]:FallGroup [Stable] | 1.61 | 0.11 | 0.9 | 2.88 |  |
| AgeGroup [60-69:SOA [150]:FallGroup [Increase] | 0.26 | .001 | 0.12 | 0.59 |  |
| AgeGroup [70+]:SOA [150]:FallGroup [Increase] | 0.18 | <.001 | 0.07 | 0.46 |  |
| AgeGroup [60-69]:SOA [230]:FallGroup [Increase] | 0.86 | 0.7 | 0.39 | 1.89 |  |
| AgeGroup [70+]:SOA [230]:FallGroup [Increase] | 0.39 | 0.05 | 0.15 | 1.02 |  |
| AgeGroup [60-69]:SOA [150]:TUGGroup [Moderate] | 1.18 | 0.43 | 0.78 | 1.8 |  |
| AgeGroup 70+:SOA [150]:TUGGroup [Moderate] | 1.24 | 0.46 | 0.7 | 2.21 |  |
| AgeGroup [60-69]:SOA [230]:TUGGroup [Moderate] | 1.2 | 0.4 | 0.79 | 1.82 |  |
| AgeGroup [70+]:SOA [230]:TUGGroup [Moderate] | 1.74 | 0.06 | 0.97 | 3.13 |  |
| AgeGroup [60-69]:SOA [150:]TUGGroup [High] | 1.07 | 0.8 | 0.61 | 1.88 |  |
| AgeGroup [70+]:SOA [150]:TUGGroup [High] | 1.74 | 0.09 | 0.91 | 3.33 |  |
| AgeGroup [60-69]:SOA [230]:TUGGroup [High] | 0.87 | 0.62 | 0.5 | 1.51 |  |
| AgeGroup [70+]:SOA [230]:TUGGroup [High] | 1.49 | 0.24 | 0.77 | 2.88 |  |

| **Table S7.**  Full results of the main model predicting accuracy on 2B1F trials of the SIFI. | | | | | |
| --- | --- | --- | --- | --- | --- |
| **Term** | **β** | ***Std. error*** | ***z*** | **Lower 95% CI** | **Upper 95% CI** |
| (Intercept) | -3.22 | 1.15 | -2.79 | -5.49 | -0.96 |
| AgeGroup [60-69] | 0.09 | 0.24 | 0.36 | -0.38 | 0.55 |
| AgeGroup [70+] | -0.27 | 0.32 | -0.83 | -0.9 | 0.37 |
| SOA [150] | -0.12 | 0.14 | -0.81 | -0.4 | 0.16 |
| SOA [230] | 0.54 | 0.14 | 3.79 | 0.26 | 0.82 |
| Sex [Female] | 0.73 | 0.11 | 6.38 | 0.51 | 0.95 |
| PrePost [Pre] | -0.22 | 0.06 | -4.03 | -0.33 | -0.12 |
| FallGroup [Decrease] | 0.27 | 0.41 | 0.65 | -0.54 | 1.08 |
| FallGroup [Stable] | 0.29 | 0.25 | 1.15 | -0.2 | 0.78 |
| FallGroup [Increase] | -0.02 | 0.27 | -0.06 | -0.54 | 0.51 |
| TUGGroup [Moderate] | 0.01 | 0.22 | 0.04 | -0.42 | 0.44 |
| TUGGroup [High] | -0.41 | 0.3 | -1.37 | -0.99 | 0.18 |
| Education [Secondary] | 0.16 | 0.16 | 0.99 | -0.16 | 0.48 |
| Education [Tertiary] | 0.22 | 0.16 | 1.31 | -0.11 | 0.54 |
| MoCA | 0.14 | 0.06 | 2.43 | 0.03 | 0.25 |
| BMI | 0.06 | 0.05 | 1.11 | -0.04 | 0.16 |
| VAS | -0.04 | 0.05 | -0.69 | -0.14 | 0.07 |
| SR_vision [Fair] | -2.18 | 0.65 | -3.37 | -3.46 | -0.91 |
| SR_vision [Good] | -2.23 | 0.62 | -3.58 | -3.45 | -1.01 |
| SR_vision [Very Good] | -2.16 | 0.62 | -3.46 | -3.38 | -0.93 |
| SR_vision [Excellent] | -1.94 | 0.63 | -3.08 | -3.17 | -0.7 |
| SR_audition [Fair] | -0.81 | 0.46 | -1.75 | -1.71 | 0.1 |
| SR_audition [Good] | -0.69 | 0.45 | -1.53 | -1.57 | 0.19 |
| SR_audition [Very Good] | -0.86 | 0.45 | -1.91 | -1.75 | 0.02 |
| SR_audition [Excellent] | -0.88 | 0.46 | -1.92 | -1.78 | 0.02 |
| HearingAid [Yes] | 0.53 | 0.22 | 2.38 | 0.09 | 0.97 |
| Cardiac [1] | 0.43 | 0.27 | 1.58 | -0.1 | 0.96 |
| Cardiac [2+] | 0.78 | 0.77 | 1.02 | -0.72 | 2.29 |
| NonCardiac [1] | 0.23 | 0.16 | 1.38 | -0.1 | 0.55 |
| NonCardiac [2+] | 0.15 | 0.58 | 0.26 | -0.98 | 1.28 |
| Psychiatric [1] | 0.34 | 0.27 | 1.25 | -0.19 | 0.87 |
| Psychiatric [2+] | 0.28 | 0.31 | 0.91 | -0.33 | 0.89 |
| Cong_1B1F [0.5] | 5.27 | 0.89 | 5.9 | 3.52 | 7.03 |
| Cong_1B1F [1] | 6.07 | 0.85 | 7.17 | 4.41 | 7.73 |
| Uni_0B2F [0.5] | -0.56 | 0.12 | -4.54 | -0.8 | -0.32 |
| Uni_0B2F [1] | -0.69 | 0.17 | -4.14 | -1.01 | -0.36 |
| Uni_2B0F [0.5] | -0.33 | 0.14 | -2.43 | -0.6 | -0.06 |
| Uni_2B0F [1] | -0.12 | 0.12 | -1.05 | -0.36 | 0.11 |
| AgeGroup [60-69]:SOA [150] | -0.27 | 0.18 | -1.55 | -0.62 | 0.07 |
| AgeGroup [70+]:SOA [150] | -1.17 | 0.26 | -4.52 | -1.68 | -0.66 |
| AgeGroup [60-69]:SOA [230] | -0.44 | 0.18 | -2.53 | -0.79 | -0.1 |
| AgeGroup [70+]:SOA [230] | -1.85 | 0.27 | -6.92 | -2.37 | -1.32 |
| SOA [150]:Sex [Female] | -1.03 | 0.09 | -11.99 | -1.2 | -0.87 |
| SOA [230]:Sex [Female] | -1.42 | 0.09 | -16.32 | -1.59 | -1.25 |
| SOA [150]:PrePost [Pre] | -0.57 | 0.08 | -6.9 | -0.73 | -0.41 |
| SOA [230]:PrePost [Pre] | -0.95 | 0.08 | -11.46 | -1.12 | -0.79 |
| SOA [150]:MoCA | 0.27 | 0.04 | 6.27 | 0.19 | 0.36 |
| SOA [230]:MoCA | 0.37 | 0.04 | 8.35 | 0.28 | 0.45 |
| SOA [150]:FallGroup [Decrease] | 0.87 | 0.31 | 2.84 | 0.27 | 1.47 |
| SOA [230]:FallGroup [Decrease] | 0.51 | 0.31 | 1.65 | -0.1 | 1.11 |
| SOA [150]:FallGroup [Stable] | 0.14 | 0.19 | 0.78 | -0.22 | 0.51 |
| SOA [230]:FallGroup [Stable] | 0 | 0.19 | 0 | -0.37 | 0.37 |
| SOA [150]:FallGroup [Increase] | -0.05 | 0.2 | -0.23 | -0.44 | 0.35 |
| SOA [230]:FallGroup [Increase] | -0.04 | 0.2 | -0.19 | -0.43 | 0.36 |
| AgeGroup [60-69]:FallGroup [Decrease] | -0.77 | 0.53 | -1.44 | -1.81 | 0.28 |
| AgeGroup [70+]:FallGroup [Decrease] | 0.53 | 0.58 | 0.91 | -0.61 | 1.67 |
| AgeGroup [60-69]:FallGroup [Stable] | -0.26 | 0.32 | -0.84 | -0.89 | 0.36 |
| AgeGroup [70+]:FallGroup [Stable] | 0.17 | 0.37 | 0.44 | -0.56 | 0.89 |
| AgeGroup [60-69]:FallGroup [Increase] | -0.11 | 0.34 | -0.33 | -0.78 | 0.55 |
| AgeGroup [70+]:FallGroup [Increase] | 0.2 | 0.4 | 0.5 | -0.58 | 0.98 |
| SOA [150]:TUGGroup [Moderate] | -0.1 | 0.16 | -0.64 | -0.42 | 0.22 |
| SOA [230]:TUGGroup [Moderate] | -0.37 | 0.16 | -2.28 | -0.69 | -0.05 |
| SOA [150]:TUGGroup [High] | -0.19 | 0.22 | -0.85 | -0.63 | 0.25 |
| SOA [230]:TUGGroup [High] | -0.19 | 0.22 | -0.87 | -0.63 | 0.24 |
| AgeGroup [60-69]:TUGGroup [Moderate] | -0.09 | 0.28 | -0.31 | -0.64 | 0.47 |
| AgeGroup [70+]:TUGGroup [Moderate] | -0.19 | 0.36 | -0.52 | -0.9 | 0.53 |
| AgeGroup [60-69]:TUGGroup [High] | 0.09 | 0.37 | 0.23 | -0.64 | 0.81 |
| AgeGroup [70+]:TUGGroup [High] | 0.33 | 0.42 | 0.8 | -0.48 | 1.15 |
| AgeGroup [60-69]:SOA [150]:FallGroup [Decrease] | -1.33 | 0.41 | -3.22 | -2.14 | -0.52 |
| AgeGroup [70+]:SOA [150]:FallGroup [Decrease] | -1.7 | 0.48 | -3.58 | -2.64 | -0.77 |
| AgeGroup [60-69]:SOA [230]:FallGroup [Decrease] | -0.16 | 0.4 | -0.39 | -0.95 | 0.64 |
| AgeGroup [70+]:SOA [230]:FallGroup [Decrease] | -0.93 | 0.48 | -1.93 | -1.88 | 0.01 |
| AgeGroup [60-69]:SOA [150]:FallGroup [Stable] | -0.43 | 0.24 | -1.79 | -0.9 | 0.04 |
| AgeGroup [70+]:SOA [150]:FallGroup [Stable] | -0.13 | 0.29 | -0.46 | -0.71 | 0.44 |
| AgeGroup [60-69]:SOA [230]:FallGroup [Stable] | -0.26 | 0.24 | -1.07 | -0.73 | 0.21 |
| AgeGroup [70+]:SOA [230]:FallGroup [Stable] | 0.47 | 0.3 | 1.59 | -0.11 | 1.06 |
| AgeGroup [60-69:SOA [150]:FallGroup [Increase] | -0.16 | 0.26 | -0.62 | -0.66 | 0.34 |
| AgeGroup [70+]:SOA [150]:FallGroup [Increase] | 0.15 | 0.31 | 0.48 | -0.47 | 0.76 |
| AgeGroup [60-69]:SOA [230]:FallGroup [Increase] | 0.02 | 0.26 | 0.07 | -0.49 | 0.52 |
| AgeGroup [70+]:SOA [230]:FallGroup [Increase] | 0.26 | 0.32 | 0.81 | -0.37 | 0.89 |
| AgeGroup [60-69]:SOA [150]:TUGGroup [Moderate] | 0.17 | 0.21 | 0.79 | -0.25 | 0.59 |
| AgeGroup 70+:SOA [150]:TUGGroup [Moderate] | 0.22 | 0.29 | 0.74 | -0.36 | 0.79 |
| AgeGroup [60-69]:SOA [230]:TUGGroup [Moderate] | 0.18 | 0.21 | 0.85 | -0.24 | 0.6 |
| AgeGroup [70+]:SOA [230]:TUGGroup [Moderate] | 0.55 | 0.3 | 1.85 | -0.03 | 1.14 |
| AgeGroup [60-69]:SOA [150:]TUGGroup [High] | 0.07 | 0.29 | 0.25 | -0.49 | 0.63 |
| AgeGroup [70+]:SOA [150]:TUGGroup [High] | 0.55 | 0.33 | 1.68 | -0.09 | 1.2 |
| AgeGroup [60-69]:SOA [230]:TUGGroup [High] | -0.14 | 0.28 | -0.5 | -0.7 | 0.41 |
| AgeGroup [70+]:SOA [230]:TUGGroup [High] | 0.4 | 0.34 | 1.18 | -0.26 | 1.06 |
